# SARS-CoV-2-infected hiPSC-derived cardiomyocytes reveal dynamic changes in the COVID-19 hearts

**DOI:** 10.1186/s13287-023-03603-1

**Published:** 2023-12-12

**Authors:** Xiao Li, Hengrui Hu, Wanlin Liu, Qiyu Zhang, Yujie Wang, Xingjuan Chen, Yunping Zhu, Zhihong Hu, Manli Wang, Jie Ma, Ling Leng

**Affiliations:** 1 Stem Cell and Regenerative Medicine Lab, Department of Medical Science Research Center, Institute of Clinical Medicine, State Key Laboratory of Complex Severe and Rare Diseases, Translational Medicine Center, Peking Union Medical College Hospital, Chinese Academy of Medical Sciences and Peking Union Medical College, Beijing, 100730 China; 2 State Key Laboratory of Proteomics, Beijing Proteome Research Center, National Center for Protein Sciences (Beijing), Beijing Institute of Lifeomics, Beijing, 102206 China; 3 State Key Laboratory of Virology, Center for Biosafety Mega-Science, Chinese Academy of Sciences, Wuhan Institute of Virology, Wuhan, 430071 China; 4 Institute of Medical Research, Northwestern Polytechnical University, Xi’an, 710072 China; 5 Basic Medical School, Anhui Medical University, Anhui, 230032 China

**Keywords:** COVID-19, SARS-CoV-2, hiPSC-derived cardiomyocytes, Heart, Proteome

## Abstract

**Background:**

The ongoing coronavirus disease 2019 (COVID-19) pandemic has had an enormous impact on our societies. Moreover, the disease’s extensive and sustained symptoms are now becoming a nonnegligible medical challenge. In this respect, data indicate that heart failure is one of the most common readmission diagnoses among COVID-19 patients.

**Methods:**

In this study, we used human induced pluripotent stem cell (hiPSC)-derived cardiomyocytes to develop an in vitro model of severe acute respiratory syndrome coronavirus 2 (SARS-CoV-2) infection and studied the dynamic changes occurring in cardiomyocytes after SARS-CoV-2 infection.

**Results:**

To this end, we have created an effective time series SARS-CoV-2 infection model exhibiting different functional patterns of up- and downregulated proteins, and demonstrating that SARS-CoV-2 mainly affects (i) the lipid and the energy metabolism of hiPSC-derived cardiomyocytes during the early infection stage, and (ii) the DNA repair ability of cardiomyocytes during the late infection stage. By analyzing the proteome changes occurring at different infection timepoints, we were able to observe that the simulated disease (COVID-19) course developed rapidly, and that each of the studied timepoints was characterized by a distinct protein expression pattern.

**Conclusions:**

Our findings highlight the importance of early detection and personalized treatment based on the disease stage. Finally, by combing the proteomics data with virus-host interaction network analysis, we were able to identify several potential drug targets for the disease.

**Supplementary Information:**

The online version contains supplementary material available at 10.1186/s13287-023-03603-1.

## Background

The coronavirus disease 2019 (COVID-19) pandemic has been raging for more than three years. By September 2023, the World Health Organization has received reports of over 6.95 million deaths resulting from confirmed cases of COVID-19. Although the lethality rate is gradually decreasing, the extensive and sustained symptoms known as “long COVID” are becoming a nonnegligible medical challenge. The possibility that the COVID-19 sequelae are associated with different factors other than the acute illness severity suggests that COVID-19 has a broader and more extensive influence [1]. With the declaration from the WHO that COVID-19 no longer constitutes a global health emergency, epidemic prevention policies are relaxing worldwide. However, given the rapid mutation of SARS-CoV-2, its high level of contagiousness, and the widespread infection, the potential long-term effects of long COVID on cardiovascular health and mortality remain a significant global concern [2, 3]. Frequently reported signs and symptoms include cardiac dysfunction, pericardial effusion or myocarditis [4]. These severe symptoms might lead to the establishment of permanent and irreversible damage. In fact, heart failure is one of the most common readmission diagnoses among COVID-19 patients [5]. Even with prior vaccination, the cardiovascular sequelae of COVID-19, including acute coronary syndrome, atrial fibrillation, heart failure, myocardial infarction, and pericarditis, can only be reduced but not completely eliminated [6].

Myocardial injury has been widely reported by postmortem studies [7–10]. Several models have been employed in the study of the COVID-19-related myocardial syndrome. Severe acute respiratory syndrome coronavirus 2 (SARS-CoV-2)-infected HFH4-hACE2 animal models were effectively generated at the onset of the outbreak, and the viral RNA was found in the eyes, the heart, and the brain of SARS-CoV-2-infected HFH4-hACE2 mice [11]. Cardiomyocytes that derived from human induced pluripotent stem cells (hiPSCs) could be used in order to construct a model of the SARS-CoV-2 infection and to investigate its cytopathic characteristics. According to the findings, the infection has altered gene expression and has accelerated the sarcomeric fragmentation. Autopsy samples have revealed myofibrillar abnormalities comparable to those reported in hiPSC-derived infected cells in the hearts of SARS-CoV-2-infected patients with confirmed myocarditis and those without cardiac involvement [12]. In a separate study, the cardiac tropism of SARS-CoV-2 in hiPSC-derived cardiomyocytes and in hiPSC-derived smooth muscle cells was investigated. The data support the hypothesis that, regardless of inflammation or coagulopathy, SARS-CoV-2 can induce a direct functional cardiac damage by either inducing cell death or by impairing electro-mechanical functions [13]. Overall, COVID-19 patients with cardiac symptoms may acquire a permanently reduced cardiac function due to the irreversible nature of the damage induced on their cardiomyocytes.

In addition, proteomic technology has been employed to study the presence of myocarditis and potential fibrosis in the heart of COVID-19 patients [14, 15]. The analysis of cardiac tissue atlases has revealed transcriptional changes in multiple types of cells within the heart tissue of COVID-19 donors [16]. When comparing the cardiac samples from patients with COVID-19 to those from the healthy individuals, both the cell composition and gene expression were found to be altered. Specifically, the proportions of cardiomyocytes and of pericytes were found to be significantly decreased [16]. However, there is a lack of time series-focused and SARS-CoV-2 infection-related research on cardiomyocytes, which is essential for understanding of the acute infection of these cells by SARS-CoV-2. In this study, we established a time series SARS-CoV-2 infection model of the hiPSC-derived cardiomyocytes, and combined it with the proteomics technology to study the dynamic changes in proteome profiles and explore the potential targets of the direct SARS-CoV-2-induced injury to cardiomyocytes.

## Methods

### hiPSC-derived cardiomyocytes differentiation and purification

The hiPSC was obtained from Nuwacell Biotechnologies Co., Ltd (RC01001-B; Female). Culture plates were coated with Matrigel (354,277; Corning) and each well was washed with Dulbecco’s PBS (1 mL; 37350; STEMCELL Technologies), and then filled with Gentle Cell Dissociation Reagent (1 mL/well; 100–0485; STEMCELL Technologies). hiPSCs with a high expression of pluripotency markers were maintained in mTeSR1 (85850; STEMCELL Technologies) in Matrigel-coated 6-well plates, and were incubated at 37 °C, 5% CO_2_. After pipetting up and down 3–4 times in order to dislodge cells, we transferred the cells to a tube and centrifuged them for 5 min at 300 × *g*. The obtained cell pellet was resuspended in mTeSR1 (1 mL) containing Y-27632 (10 μM; 72302; STEMCELL Technologies). hiPSC were then added into the culture plates with mTeSR1 (1 mL/well) containing Y-27632 (10 μM) and were incubated at 37 °C for 24 h. Subsequently, the medium was replaced with mTeSR1 (1 mL/well) and the cells were incubated at 37 °C for further 24 h. The medium in each well was then replaced with STEMdiff Ventricular Cardiomyocyte Differentiation Medium A (2 mL; 1:100 dilution; 05012; STEMCELL Technologies) containing 20 μL of Matrigel (Corning), and the cells were incubated at 37 °C for 48 h. After that, the medium in each well was replaced with STEMdiff Ventricular Cardiomyocyte Differentiation Medium B (2 mL; 05013; STEMCELL Technologies) on day 2, and with STEMdiff Ventricular Cardiomyocyte Differentiation Medium C (2 mL; 05014; STEMCELL Technologies) on days 4 and 6. Subsequently, the medium in each well was replaced with STEMdiff Cardiomyocyte Maintenance Medium (2 mL; 05015; STEMCELL Technologies) every 2 days, for 4 times. Finally, the hiPSC-derived cardiomyocytes were harvested for use in the virus infection experiment.

### SARS-CoV-2 infection experiment

The hiPSC-derived cardiomyocytes were infected with SARS-CoV-2 (strain: nCoV-2019BetaCoV/Wuhan/ WIV04/2019) [17] at a multiplicity of infection (MOI) of 1. Briefly, the cells were incubated with the virus for 2 h, and the culture medium was then replaced with fresh medium. The infected cells were collected at 24, 48, and 72 h post-infection, respectively. For the undertaking of protein extraction, the cells were lysed by lysis buffer (2% SDS, 100 mM Tris–HCl, pH 8.0), the obtained lysates were incubated in a metal bath at 90 °C for 30 min, and their supernatants were then collected after centrifugation at 15,000 × *g* for 15 min. All infection-related experiments were carried out in a BSL-3 lab.

### Cytopathological staining and analysis

hiPSC-derived cardiomyocytes were fixed in 4% formaldehyde for 20 min, and were then washed in PBS. Subsequently, the cells were treated with 0.25% Triton X-100 for 20 min, at 26 °C. The cells were then incubated with primary antibodies, overnight, at 4 °C. After an incubation for 1 h at 26 °C with secondary antibodies and counterstaining with DAPI, the cells were sealed with Fluoro-Gel. Negative control samples were incubated with secondary antibody alone. Pictures were taken at × 20/ × 40 magnification by using a confocal microscope.

### Extraction of the SARS-CoV-2 viral particle proteins

Vero E6 cells were infected with SARS-CoV-2 at an MOI of 0.001, and the infected cell supernatant was collected at 48 h post-infection and clarified via centrifugation at 5,000 rpm for 5 min. In total, 50 mL of the infected cell supernatant was concentrated by a 100-kDa cut-off Centricon® Plus-70 centrifugal filter (Millipore) to a final volume of 1 mL. The viral particles were then added into the same volume of 2 × protein lysis buffer (4% SDS, 200 mM Tris–HCl, pH 8.0) and were incubated in a metal bath at 90°C for 30 min. The viral protein-containing supernatant was then collected after a 13,000-rpm centrifugation at room temperature for 15 min.

### Protein extraction, digestion, and LC–MS/MS analysis

We diluted 100 μg of the cell samples into a 4 × volume of cold acetone, mixed well, and allowed the proteins to precipitate at − 20 °C, overnight. We then centrifuged the samples at 14,000 × *g* for 10min, added 1 mL of cold acetone in order to wash the pellet, allow the proteins to precipitate once again, and then dried in air. Subsequently, we added 100 μl of 8 M urea, centrifuged at 14,000 × *g* for 20 min, and obtained the supernatant, sub package. A 100-µg aliquot of the extracted proteins from each sample was then subjected to reduction by adding 200 mM of dithiothreitol solution and incubating at 37 °C for 1 h. The sample was then diluted 4 times by adding a 25-mM ammonium bicarbonate buffer. Trypsin was then added (at a trypsin to protein ratio of 1:50), and the samples were incubated at 37 °C, overnight. The next day, we added 50 μL of 0.1% formic acid to each sample in order to terminate the digestion.

For the spectral library construction, shotgun proteomics analyses were performed by using an EASY-nLC 1200 ultra-high-performance pressure system coupled with a Q Exactive HF-X mass spectrometer (Thermo Fisher Scientific), operating in the data-dependent acquisition (DDA) mode. Briefly, the mass spectrometer was operated in a “Top-40” data-dependent mode, thereby collecting MS spectra in the Orbitrap mass analyzer (120,000 resolution; 350–1,500 m/z range) with an automatic gain control (AGC) target of 3E6 and a maximum ion injection time of 80 ms. The most intense ions from the full scan were isolated with an isolation width of 1.6 m/z. Following higher-energy collisional dissociation with a normalized collision energy (NCE) of 27, the MS/MS spectra were collected in the Orbitrap (15,000 resolution) with an AGC target of 5E4 and a maximum ion injection time of 45 ms. The precursor dynamic exclusion was enabled with duration of 16 s.

The data-independent acquisition (DIA) scan mode was used for cell samples. The MS1 resolution was set at 60,000 (at 200 m/z), and the MS2 resolution was set at 30,000 (at 200 m/z). The m/z range covered from 350 to 1,500 m/z, and was separated into 42 acquisition windows (isolation windows varying from 14 to 312 m/z). The full scan AGC target was set at 3 × 10^6^, with an injection time of 80 ms. The DIA settings included an NCE of 25.5%, 27%, and 30%, a target value of 1 × 10^6^, and an automatic maximum injection time that was set to automatic in order to allow for the MS to continuously operate in the parallel ion filling and detection mode.

### Protein identification and quantification

The MS data of the fractionated pools (DDA MS data; three fractions) and the single-shot subject samples (DIA MS data) were used in order to generate a hybrid library in the Spectronaut software (version 15.7.220308.50606; Biognosys). The hybrid spectral library was used in order to search the MS data of the single-shot samples in the Spectronaut software for the final protein identification and quantitation. All searches were performed against the human UniProt reference proteome of canonical and isoform sequences, with 75,093 entries downloaded in August 2020. Searches used carbamidomethylation as a fixed modification and the acetylation of the protein *N*-terminus as well as the oxidation of methionine as variable modifications. Default settings were used for other parameters. In brief, a trypsin/P proteolytic cleavage rule was used, thereby permitting a maximum of two miss cleavages and a peptide length of 7–52 amino acids. Protein intensities were normalized by using the “Local Normalization” algorithm in Spectronaut, based on a local regression model. Spectral library generation has stipulated a minimum of three fragments per peptide, and maximally, the six best fragments were included. A protein and a precursor FDR of 1% were used, and the protein quantities were reported in samples only if the protein has passed the filter.

The MS/MS raw files generated from the SARS-CoV-2 viral particle proteins were analyzed using MaxQuant software (version 1.6.5.0) [18] by searching against a database containing the SwissProt human sequences and SARS-CoV-2 protein sequence database (downloaded from National Center for Biotechnology Information on June 6, 2020). Peptides were identified using a precursor mass tolerance of ≤ 4.5 ppm and a fragment mass tolerance = 20 ppm. Cysteine carbamidomethylation was set as the fixed modification, and *N*-terminal acetylation and methionine oxidation served as variable modifications. Two or fewer missed cleavages were allowed, and trypsin was set as the reference enzyme. Automatic target and reverse database searches were enabled with a maximum false discovery rate of 0.01 for peptide and protein identification.

### Statistical and bioinformatics analysis

General data transformation and statistical analysis was performed by using the *R* package software (version 4.1.2). The proteins with at least two unique peptide identifications were kept for further analysis. The quantification values of the identified proteins were normalized by taking the fraction of the total, followed by a multiplication by 10^6^ and a log2 transformation. Pairwise comparisons in order to identify proteins whose expression was significantly different between the SARS-CoV-2 infected and the control samples were performed by using the R package Limma (version 3.50.3). If the Benjamini–Hochberg-adjusted *p*-value was found to be < 0.05, then the differences were considered as statistically significant. The proteins that met the fold-change threshold were considered as upregulated (log_2_FC > 0.585) or downregulated (log_2_FC <  − 0.585); the fold change (FC) corresponds to the ratio of SARS-CoV-2-infected samples to uninfected control samples. One-way ANOVA was used in order to determine whether there were any statistically significant differences in the normalized intensities among proteins identified in hiPSC-derived cardiomyocytes after different infection times. Proteins with the one-way ANOVA *p* < 0.05 and |log_2_FC|> 0.263 were considered as specifically over- / under-expressed. The specific *p*-value is indicated in the figure legend.

The online tool DAVID (https://david.ncifcrf.gov/) [19] was used in order to annotate the proteins according to Gene Ontology biological processes analysis. The principal coordinate analysis (PCoA) of the proteins with valid values in each sample was performed by using the R package ape (version 5.5). The protein–protein interactions were retrieved from the STRING database (https://string-db.org/) [20], and the network was built by using Cystoscope (version 3.8.2) [21]. The ComplexHeatmap package (version 2.10.0) was used in order to reveal the specificity of over- and under-expressed proteins in the SARS-CoV-2 infected hiPSC-derived cardiomyocytes at the different timepoints assessed. The *R* circlize package (version 0.4.13) [22] was used in order to circularly visualize the upregulated and the downregulated proteins that are involved in multiple biological processes.

## Results

### Proteome profile reveals the dysfunction of heart tissues due to COVID-19

**Fig. 1:**
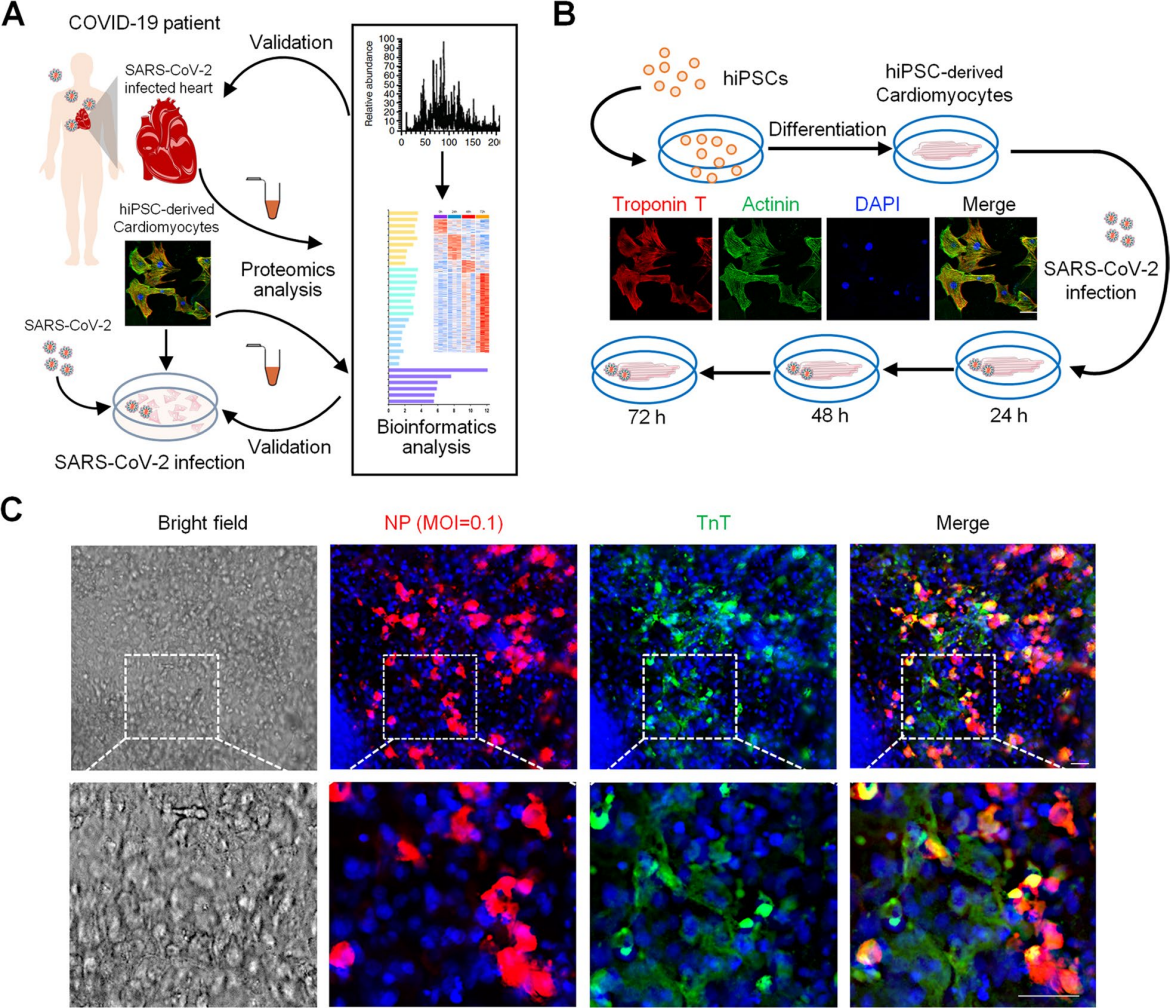
Establishment of the in vitro hiPSC-derived cardiomyocyte infection system. **A** Workflow of proteomics analysis of SARS-CoV-2-infected heart tissues and hiPSC-derived cardiomyocytes. The proteomic data of the COVID-19 patient heart tissues were obtained from a previous study [14] and were presented in supplementary Figure S1. After the induction of the SARS-CoV-2 infection, the hiPSC-derived cardiomyocytes’ proteome was analyzed by LC–MS/MS. **B** Workflow of the hiPSC-derived cardiomyocyte-based time series dynamic monitoring; hiPSC-derived cardiomyocytes were stained positive for TnT (red), *α*-actinin (green), and DAPI (blue) in order to distinguish the differentiated cardiomyocytes from iPSC (scale bar: 60 μm). **C** Immunofluorescence of the viral NP and of the cardiomyocyte marker (TnT) in SARS-CoV-2-infected hiPSC-derived cardiomyocytes at 72 h (scale bar: 50 μm)

In our previous study, a spatial region-resolved proteome map from the four regions of the heart was produced for patients with COVID-19 and normal controls [14]. In order to investigate the direct damage caused by the SARS-CoV-2 infection on human heart on a whole level, we first reanalyzed the proteome profile of tissue samples generated by the four regions of hearts from patients with COVID-19 and normal controls in our previous study [14] (Additional file 2: Table S1); then, we used the hiPSC-derived cardiomyocytes as an in vitro model to study the dynamic proteome changes during SARS-CoV-2 infection (Fig. 1A). The PCoA graph revealed the significant differences between the COVID-19 and the control groups (Additional file 1: Figure S1A). A total of 129 upregulated and 303 downregulated differentially expressed proteins (DEPs) were discovered (Additional file 1: Figure S1B, Additional file 3: Table S2), and a functional annotation was applied (Additional file 1: Figure S1C).

The functional annotation revealed that, in the COVID-19 samples, the upregulated proteins are generally enriched in the biological processes related to responses to a xenobiotic stimulus, chronic inflammatory response, neutrophil aggregation, innate immune response, etc., thereby indicating that most of the upregulated proteins are generated due to the immune response of the heart tissues to the SARS-CoV-2 infection. We have also found that energy metabolism-associated proteins are upregulated in the COVID-19 patient heart tissues. These proteins are involved in processes such as the fatty acid beta-oxidation (HADHB, HADHA, ECH1, and HSD17B4) and the electron transport coupled proton transport (MT-ND5 and MT-CO1), indicating that the hearts of patients with COVID-19 may consume excessive energy as a result of the SARS-CoV-2 infection. Moreover, apoptosis-associated proteins were found to be upregulated in the COVID-19 patient heart tissues. These proteins either act as positive regulators of cell death (HBG1, HBB, HBD, and HBA1) or are involved in the cellular response to tumor necrosis factor (FABP4, GPD1, THBS1, ASS1, and PCK2), suggesting that apoptosis may be an important cause of heart failure in patients with COVID-19.

On the other hand, proteins involved in mRNA-associated biological processes, such as mRNA splicing and transport (DDX39B, SRSF1, HNRNPA3, HNRNPA2B1, QKI, and FMR1), and nucleus response-associated processes, such as nucleosome assembly and chromosome condensation (H3-3A, H1-5, H1-4, H1-3, and H1–10), were found to be downregulated in the COVID-19 patient heart tissues, indicating that the ability of transcription in the COVID-19 patient heart tissues may be affected. Tissue skeleton-associated proteins including proteins involved in collagen fibril organization (CRTAP, TNXB, COL1A2, COL5A1, LUM, COL14A1, PXDN, DPT, and FMOD) and elastic fiber assembly (MFAP4, TNXB, LTBP4, and FBLN5) were found to be downregulated in the COVID-19 patient heart tissues. More specifically, we have found that myocardial development-associated proteins including those involved in sarcomere organization (CSRP3, SYNPO2L, LDB3, and MYPN) and muscle cell differentiation (SPEG, QKI, and H3-3A) were downregulated in the COVID-19 patient heart tissues, indicating the structure of the heart may be affected. These results suggest that the SARS-CoV-2 infection affects the biological characteristics and pathological features of heart tissues.

### Dynamic changes in proteins in a time series SARS-CoV-2 infection model

To further investigate the main effect of the SARS-CoV-2 infection on heart function, a stable cell culture model of hiPSC-derived cardiomyocytes was established (Fig. 1B). The proportion of hiPSC- derived cardiomyocytes is about 90% after selection by the method of flow cytometry (Additional file 1: Figure S2A–B). Subsequently, we examined and evaluated the parameters of the time series SARS-CoV-2 infection model (Fig. 1B). Our experiments examined the behavior of different MOIs. The number of copies of the virus at 24, 48, and 72 h post-infection showed no significant differences between the MOIs of 0.1 and 1 (Additional file 1: Figure S2C). Consequently, the 0.1-MOI condition was selected for the undertaking of the time series SARS-CoV-2 infection experiments. Immunofluorescence results revealed that the hiPSC-derived cardiomyocytes were infected, as the fluorescence staining of troponin T (TnT) and that of the nucleocapsid protein (NP) of the virus were found to be highly co-localized, and the infection efficiency of SARS-CoV-2 in hiPSC-derived cardiomyocytes at 72h was about 42.9% (Fig. 1C). Thereby, it demonstrated that the hiPSC-derived cardiomyocyte culture system was suitable for characterization of the SARS-CoV-2 infection process in cardiomyocytes.

**Fig. 2:**
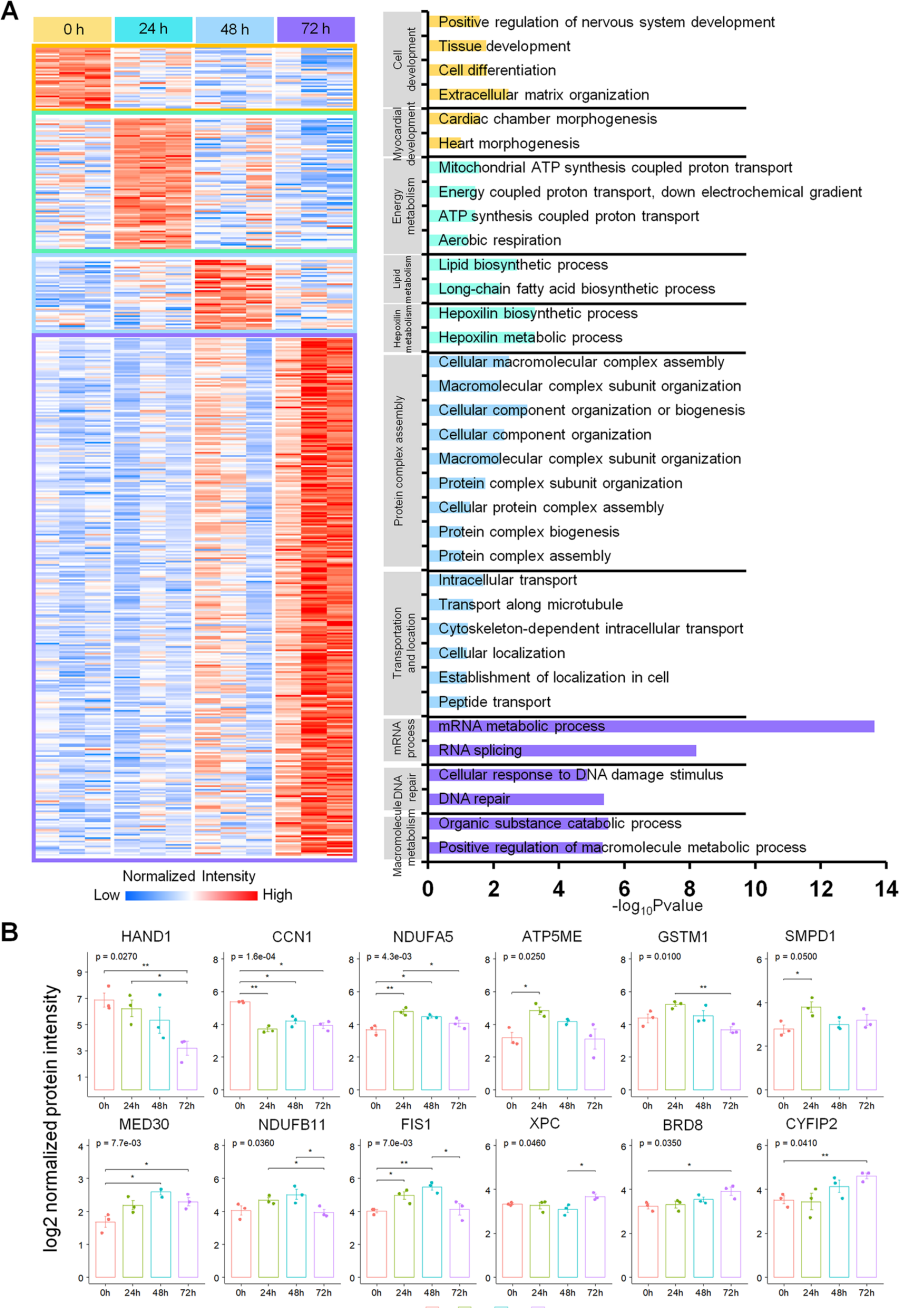
Functional annotation of the proteins found to be specifically over-expressed in the SARS-CoV-2-infected hiPSC-derived cardiomyocytes at different timepoints. **A** According to the assessed timepoints of the over-expression protein enrichment, four patterns were identified as elevated separately at 0 (marked as yellow), 24 (light green), 48 (blue), and 72 (purple) h after the induction of the SARS-CoV-2 infection; the functional annotation of the corresponding proteins was revealed (marked separately by corresponding colors); differences at an one-way ANOVA *p* < 0.05 were regarded as statistically significant in the specific region; red and blue boxes indicate proteins with increased and decreased abundance, respectively. **B** Protein expression levels of HAND1, CCN1, NDUFA5, ATP5ME, GSTM1, SMPD1, GSTP1, MED30, NDUFB11, FIS1, XPC, BRD8, and CYFIP2 in the DIA identification groups according to the normalized protein intensity; *, *p* < 0.05; **, *p* < 0.01; ***, *p* < 0.001

hiPSC-derived cardiomyocytes were then infected with SARS-CoV-2 for 24, 48, and 72 h. Samples were collected and digested for the undertaking of proteome identification (Additional file 1: Figure S3A,B, Additional file 4: Table S3). There were 354, 545, and 746 proteins that differentially expressed in hiPSC-derived cardiomyocytes with SARS-CoV-2 infection for 24, 48, and 72 h, compared to the control groups (Additional file 1: Figure S3C, Additional file 5: Table S4); among them, a total of 24 and 35 proteins are both upregulated and downregulated in COVID-19 heart tissues and infected hiPSC-derived cardiomyocytes, respectively (Additional file 1: Figure S3E). Biological function analysis showed that the upregulated proteins were enriched in the functions of apoptosis process, energy metabolism, and viral entry into host cell, while the downregulated proteins were enriched in mRNA processing and myocardial development (Additional file 1: Figure S3D). Then, the DEPs of the different timepoints were identified through an AVOVA analysis. The results have revealed specific highly-expressed protein patterns of the different timepoints assessed (Fig. 2A, Additional file 6: Table S5). Four patterns were found to be elevated separately at 0, 24, 48, and 72 h after the SARS-CoV-2 infection. The functional annotation of these proteins revealed the proteomic signatures of these patterns. At 0 h post-infection (control group), the upregulated proteins were enriched in function of cell development (through proteins such as HAND1 and CCN1, Fig. 2B); a pattern that was lost in the SARS-CoV-2 infected hiPSC-derived cardiomyocytes. As far as the 24-h post-infection upregulated pattern is concerned, the enrichment functions were associated with energy (through proteins such as NDUFA5 and ATP5ME, Fig. 2B), lipid or hepoxilin metabolism (through proteins such as GSTM1 and SMPD1, Fig. 2B), probably due to the stress response to cellular hypoxia and the energy supply in the primary infection. The occurrence of a macromolecular assembly (through proteins such as MED30 and NDUFB11, Fig. 2B) and cellular localization (through proteins such as FIS1, Fig. 2B) seems to occur as part of the 48-h post-infection pattern, thereby suggesting that the cells experience enormous energy expenditure. Finally, the mRNA process, DNA repair (through proteins such as XPC and BRD8, Fig. 2B) and the macromolecule metabolism (through proteins such as CYFIP2, Fig. 2B) were found to be enriched in the case of the 72-h post-infection pattern of upregulated proteins, thereby indicating that the SARS-CoV-2-infected hiPSC-derived cardiomyocytes were at that point of time actively rescuing and self-repairing. These results reveal the landscape of the dynamic response of cardiomyocytes after a viral infection.

**Fig. 3:**
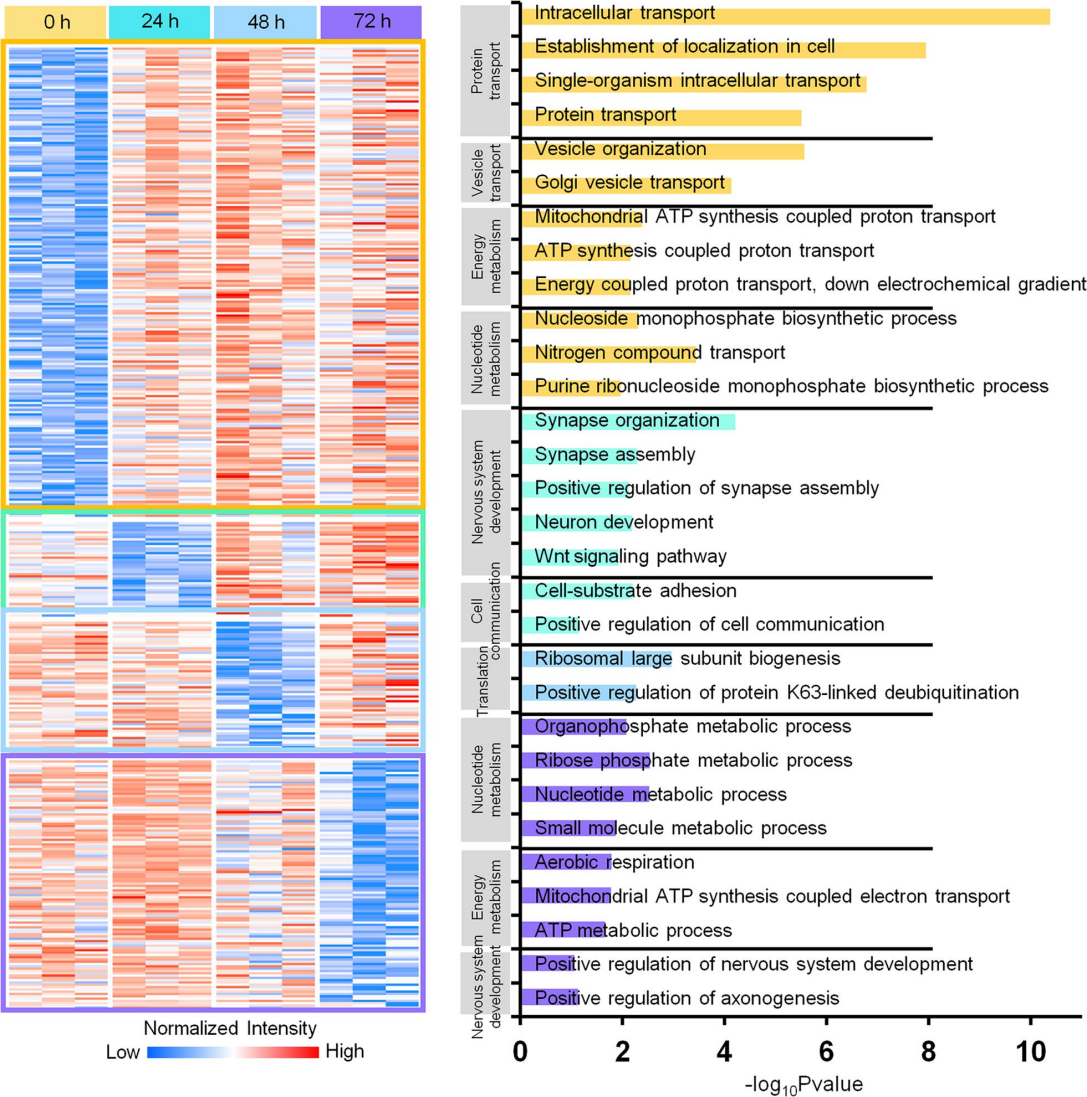
Functional annotation of the proteins specifically under-expressed in the SARS-CoV-2-infected hiPSC-derived cardiomyocytes at different timepoints. According to the assessed timepoints of the under-expression protein enrichment, four patterns were identified as elevated separately at 0 (marked as yellow), 24 (light green), 48 (blue), and 72 (purple) h after the induction of the SARS-CoV-2 infection; the functional annotation of the corresponding proteins was revealed (marked separately by corresponding colors); differences at an one-way ANOVA *p* < 0.05 were regarded as statistically significant in the specific region; red and blue boxes indicate proteins with increased and decreased abundance, respectively

Similarly, we obtained the specific under-expressed protein patterns via an expression profile clustering (Fig. 3, Additional file 7: Table S6). The downregulated proteins in the SARS-CoV-2-infected hiPSC-derived cardiomyocytes were found to be enriched in the functions of protein and vesicle transport, energy metabolism, and nucleotide metabolism, when compared to control. Among them, the abnormal upregulation of the energy metabolism process after a SARS-CoV-2 infection was found in both autopsy-derived heart tissues and SARS-CoV-2-infected hiPSC-derived cardiomyocytes, thereby further indicating that cardiomyocytes consume excessive energy after infection. Subsequently, we analyzed the specific lowly-expressed protein patterns against the different post-infection timepoints assessed (Fig. 3). Our results have shown that the downregulated proteins in the SARS-CoV-2 group at 24 h post-infection were mainly enriched in biological processes such as the nervous system development (particularly for neuronal development and synapse assembly), as well as the cell communication. Translation-associated proteins were found to be downregulated at 48 h post-infection, while nucleotide metabolism- and energy metabolism-associated proteins were found to be mainly downregulated at 72 h post-infection in the SARS-CoV-2 group (Fig. 3). The functional annotations of the proteomic data indicate that, in the early stages of SARS-CoV-2-infected hiPSC-derived cardiomyocytes, functions related to the nervous system development and cell–cell interactions are diminished, followed by translation. As the duration of the infection increases, the expression of nucleotide metabolism- and energy metabolism-related proteins decreases, thereby indicating that the cardiomyocytes may be damaged by SARS-CoV-2. These findings map the main pathways that the SARS-CoV-2 attacks over time during the development of COVID-19.

### Key molecular events of the virus-host interactions

**Fig. 4:**
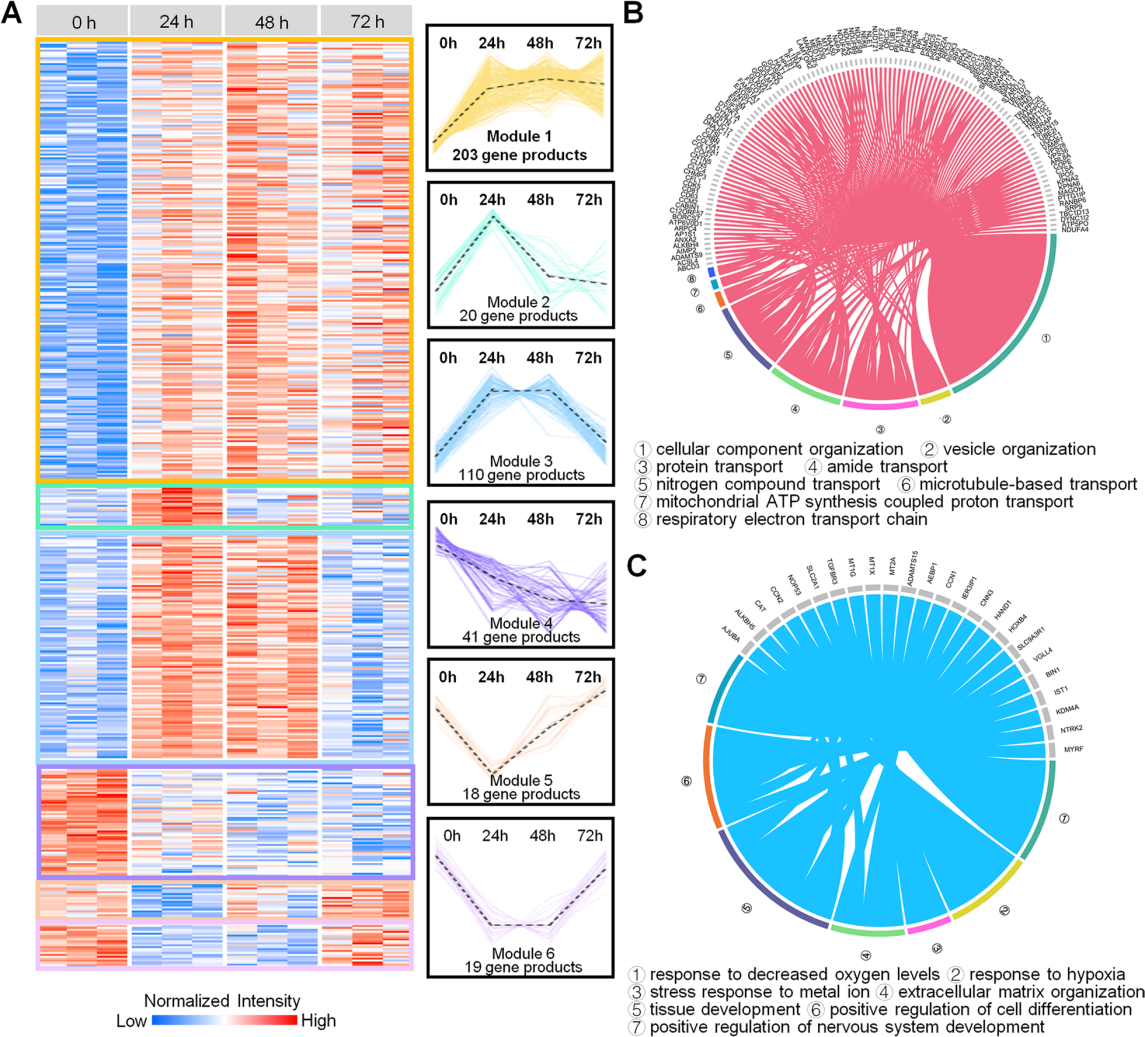
Co-expression profiles analysis of proteins identified the SARS-CoV-2-infected hiPSC-derived cardiomyocytes at different timepoint. **A** Co-expression analysis revealed six modules according to the obtained time series protein expression profiles; red and blue boxes indicate proteins with increased and decreased abundance, respectively. **B**, **C** The circos plot presents gradually over- (B, module 1 in A) and under- (C, module 4 in A) expressed proteins that are involved in multiple biological processes; terms with different colors represent different functional annotations

In an attempt to further explore the molecular events of the viral pathology in cardiomyocytes, we employed the expression trends analysis in order to obtain an insight into the temporal behavior of the COVID-19-associated time series SARS-CoV-2 infection model. The analysis revealed six modules (Fig. 4A, Additional file 8: Table S7). Gradually over-expressed proteins with the passing of the SARS-CoV-2 infection time were enriched in various organization processes (such as the cellular component organization or the vesicle organization) as well as in various transport-related processes (including protein transport, amide transport, nitrogen compound transport and so on) (Fig. 4B), thereby indicating that the SARS-CoV-2 infection continues to affect the protein and small molecule composition of host cells (an effect that may be useful for the accommodation of the viral replication packaging). Correspondingly, gradually under-expressed proteins were enriched in several response processes (such as the response to decreased oxygen levels or hypoxia), thereby indicating that the SARS-CoV-2 infection affects the oxygen levels in cardiomyocytes. In addition, the positive regulation of cell differentiation and the nervous system development processes were found to be enriched by downregulated proteins. Moreover, processes related to the extracellular matrix organization and tissue development were also found to be enriched with downregulated proteins (Fig. 4C). These results shed more light on the functional dynamics of cardiomyocytes with the increase of infection time, including the activation of various transport processes and the weakened responsiveness to hypoxia.

**Fig. 5:**
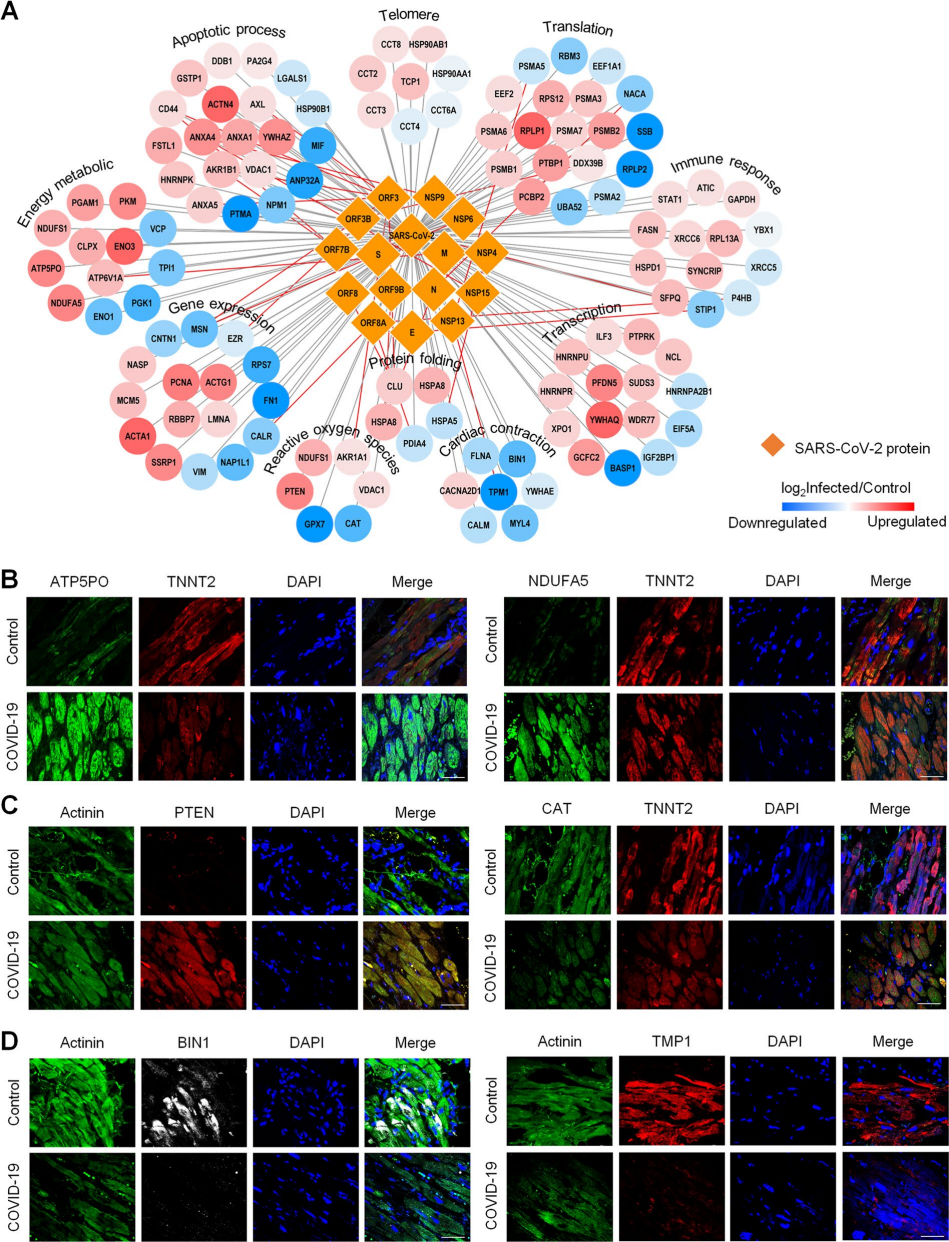
Key molecular events involved in the identified virus-host interactions. **A** Interaction network between DEPs and SARS-CoV-2 proteins; a map of the functional categories was used for the primary biological process analyses; yellow diamonds represent the structural, non-structural, and additional open reading frame proteins of SARS-CoV-2 extracted and identified from the cells, the gray lines represent the interactions between the viral proteins and DEPs, and the red lines represent the interactions reported in the previous study [29–31]. Immunofluorescence of the ATP energy metabolism related proteins ATP5PO and NDUFA5 **B**, the ROS function related proteins PTEN and CAT **C**, and the cardiac conduction function related proteins BIN1 and TMP1 **D**, combined with the cardiomyocyte marker (Actinin or TNNT2) in cardiomyocytes of COVID-19 hearts and normal controls (Scale bar: 50 µm)

In order to further investigate the functions of cardiomyocytes directly affected by viral proteins, an integrated interactome network was constructed between the viral proteins and the dynamically changed proteins with infection (Fig. 5A). Our results have shown that most of the apoptosis-related proteins (such as ANXA4, ANXA1, ANXA5, AXL, YWHAZ, and FSTL1) can interact with viral proteins (such as NSP9, NSP15, ORF3, and ORF7B), thereby indicating that the SARS-CoV-2 infection can accelerate the death of cardiomyocytes. We have also found that many proteins related to gene expression (such as PCNA, ACTG1, LMNA, RBBP7, and SSRP1), transcription (such as PFDN5, SUDS3, YWHA1, and WDR77), translation (such as RPLP1, RPS12, PSMA7, PSMB2, and PSMA3), and protein folding (such as CLU and HSPA8) can interact with viral proteins (such as N, NSP4, NSP6, NSP13, ORF3, ORF3B, ORF8, ORF8A, and ORF9B), thereby indicating that the SARS-CoV-2 can complete the viral replication and transcription process together with these proteins, and that these proteins can be used as potential antiviral drug targets. In addition, we found that viral proteins can directly affect energy metabolism, reactive oxygen species (ROS), and immune response processes, thereby indicating that these SARS-CoV-2 proteins may trigger abnormal metabolism in cardiomyocytes or cause tissue inflammation through direct interaction. These findings highlight a group of host proteins that can interact with SARS-CoV-2 proteins during the infection of cardiomyocytes and may lead to heart failure in the SARS-CoV-2 infection of cardiomyocytes. In addition, we performed immunofluorescence staining on the heart tissue of patients with COVID-19, which could validate our findings in cardiomyocytes. As shown in Fig. 5B–C, the ATP function related proteins ATP5PO and NDUFA5 are upregulated, the ROS-related proteins PTEN and CAT are upregulated and downregulated, respectively, in the cardiomyocytes of COVID-19 hearts compared with the control samples, indicating that energy metabolism was abnormally active after infection. Further, we validated the specific proteins involved in functions of cardiac conduction, which is associated with beating frequency, by immunofluorescence staining experiments. As shown in Fig. 5D, the cardiac conduction related proteins BIN1 and TPM1 are downregulated in the cardiomyocytes of COVID-19 hearts compared with the control samples, suggesting the virus infection could cause a serious impact on the systolic and diastolic functions of heart. All these results presented in our study provide molecular evidence to prove that SARS-CoV-2 infection may lead to cardiac complications.

## Discussion

In this study, we have developed a hiPSC-derived cardiomyocytes infection model for SARS-CoV-2. The development of the time series infection model was proved to be effective. Different functional patterns of up- and downregulated proteins were discovered, and when combined with our findings in COVID-19 patients heart tissues, they revealed the existence of distinct infection phases.

**Fig. 6:**
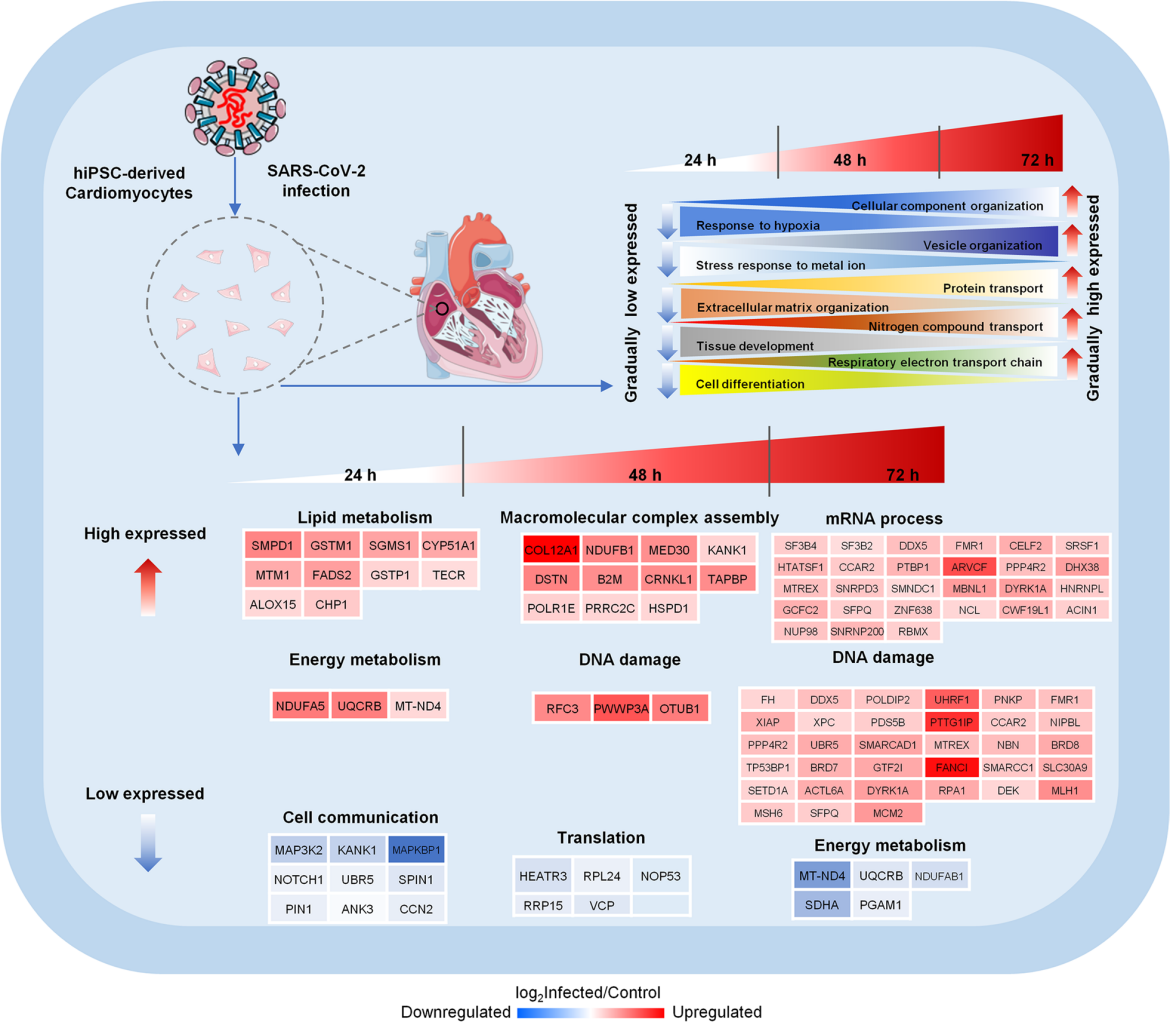
The dynamic model of the hiPSC-derived cardiomyocytes’ proteome reveals functional changes during a SARS-CoV-2 infection. According to the dynamics of the protein expression abundance under certain temporal conditions, the DEPs can be divided into gradually and specifically DEPs, and the functional enrichment of the different patterns observed has been analyzed separately; the colors of the protein nodes indicate the measured log_2_ fold-change of the proteins expressed in SARS-CoV-2-infected and control hiPSC-derived cardiomyocytes; red and blue boxes indicate proteins with up- and downregulated abundance, respectively

Based on the dynamic of protein expression abundance under certain temporal conditions, the DEPs can be divided into gradually/specifically DEPs and can be analyzed for functional enrichment separately. The proteins that gradually over-expressed over time were mainly involved in cellular component organization, vesicle organization, protein transport, nitrogen compound transport, as well as respiratory electron transport chain; these processes demonstrate the cardiomyocyte response to the SARS-CoV-2 infection. On the other hand, the proteins found to be gradually under-expressed were associated with functions of the response to hypoxia, the stress response to metal ions, the extracellular matrix organization, tissue development, and cell differentiation; these processes represent vital cellular physiological functions of the cardiomyocytes that have been gradually disrupted by the SARS-CoV-2 (Fig. 6).

Specifically over-expressed proteins represent the core functionality of the particular stage of infection. Lipid metabolism is mainly a function affected at the 24-h upregulated protein pattern, and the probable reason for that could be the cardiomyocyte’s stress response to the energy supply. The occurrence of a macromolecular complex assembly at the 48-h pattern may suggest a level of repair attempted on behalf of the cardiomyocytes. Finally, the mRNA process was found enriched at the 72-h pattern, thereby implying that the translation of proteins could be a response to the infection (Figs. 2 and 6). The dynamic of the over-expressed proteins’ functional enrichment across the time series of the infection implies that the cells are actively attempting to survive and self-repair. It demonstrates a landscape of dynamic responses of the cardiomyocytes over time after a viral attack. On the other hand, the functional enrichment of specifically under-expressed proteins reveals a process of progressive virus-induced damaged upon the cardiomyocytes. In the early stages of SARS-CoV-2-infected hiPSC-derived cardiomyocytes, cell communication has been found impaired, followed by translation. As the duration of the infection is prolonged, the expression of energy metabolism-related proteins decreases, indicating that the cells may be damaged (Figs. 3 and 6). Our findings also demonstrate the main features of the pathways that the virus attacks over time during the development of COVID-19. In addition, energy metabolism-related proteins were found to be up- and downregulated, respectively, thereby reflecting that this process is–to a large extent–dynamic, and affects core physiological functions of the infected cardiomyocytes.

The persistent symptoms of long-term sequelae following SARS-CoV-2 infection has emerged as a major concern [23–25]. In previous research, we applied sera proteomics in the active and recovered COVID-19 patients, as well as healthy individuals to discover the biomarkers COVID-19 in sera. By comparing with the DEPs of SARS-CoV-2-infected hiPSC-derived cardiomyocytes with the sera proteome profile obtained in this study, several proteins (e.g., FN1, NCAM1, and OGN) were also identified in the sera samples, providing further evidence for these proteins as potential diagnostic or prognostic biomarkers for COVID-19. Captur et al*.* performed the plasma proteome analysis of 156 healthcare workers with and without lab-confirmed SARS-CoV-2 infection, to correlate the expression levels of 91 proteins panel with the long COVID symptoms [26]. By comparing with this study, a dozen proteins that differentially expressed in the SAR-CoV-2 infected hiPSC-derived cardiomyocytes were associated with the persistent symptoms after acute COVID-19. These proteins mainly involved in the biological process of acute inflammatory response (e.g., SERPINA1, SERPINA3, and CRP), regulation of hydrolase activity (e.g., COL6A3, CTSB, and C4A), reactive oxygen species metabolic process (e.g., CRP, SOD3, and BST1), etc. In summary, the proteins which are dynamically changed in the process of SAR-CoV-2 infection of hiPSC-derived cardiomyocytes, could be associated with important physiological functions of cardiomyocytes; further research on these proteins would be helpful for understanding the long-term effects of COVID-19.

## Conclusion

After analyzing the time-dependent variations observed in the SARS-CoV-2-infected hiPSC-derived cardiomyocytes, we discovered that the disease course develops rapidly, and that different periods are characterized by distinct expression patterns. This fact suggests that the early detection and the administration of a personalized treatment according to the specific stage of the disease would be advantageous, and that specific drug targets for the treatment of COVID-19 should be explored according to the disease stage. We have, herein, purified viral particles in hiPSC-derived cardiomyocytes and have identified human proteins on these particles. These protein identifications are very likely to represent the host proteins that directly involved in the SARS-CoV-2 replication and packaging. Also, these proteins should be considered as important elements for the discovery and development of host protein-targeting antiviral drugs.

### Supplementary Information

**Additional file 1. **Supplementary Figures.

**Additional file 2. Table S1.** All protein identified in COVID-19 and control heart tissue samples.

**Additional file 3. Table S2.** Differentially expressed proteins identified in heart tissues between patients with COVID-19 and control samples.

**Additional file 4. Table S3.** All proteins identified in hiPSC-derived cardiomyocytes with different infection timepoints.

**Additional file 5. Table S4.** Differentially expressed proteins identified in SARS-CoV-2-infected hiPSC-derived cardiomyocytes at 24, 48, and 72 h.

**Additional file 6. Table S5.** Detailed list of specifically overexpressed proteins in the SARS-CoV-2-infected hiPSC-derived cardiomyocytes at different timepoints.

**Additional file 7. Table S6.** Detailed list of specifically under-expressed proteins in the SARS-CoV-2-infected hiPSC-derived cardiomyocytes at different timepoints.

**Additional file 8. Table S7.** Detailed list of proteins in six expression profiles identified by co-expression analysis.

## Data Availability

All proteomics data generated from hiPSC-derived cardiomyocytes have been deposited to the ProteomeXchange Consortium via iProX [27, 28] repository with identifier IPX0005447000 / PXD047427.

## Abbreviations

AGC: Automatic gain control
COVID-19: Coronavirus disease 2019
DDA: Data-dependent acquisition
DEP: Differentially expressed protein
DIA: Data-independent acquisition
FDR: False discovery rate
hiPSCs: Human induced pluripotent stem cells
MOI: Multiplicity of infection
NCE: Normalized collision energy
NP: Nucleocapsid protein
PBS: Phosphate-buffered saline
PCoA: Principal coordinate analysis
ROS: Reactive oxygen species
SARS-CoV-2: Severe acute respiratory syndrome coronavirus 2
TnT: Troponin T
