# Supplementary material for: SARS-CoV-2-infected hiPSC-derived cardiomyocytes reveal dynamic changes in the COVID-19 hearts
Source: Stem Cell Res Ther. 2023 Dec 12;14:361. doi: 10.1186/s13287-023-03603-1 (PMC10717444; doi:10.1186/s13287-023-03603-1)
Supplement: Supplementary file 1 — Additional file 1. Supplementary Figures. [file 13287_2023_3603_MOESM1_ESM.pdf]

Supplementary Figures

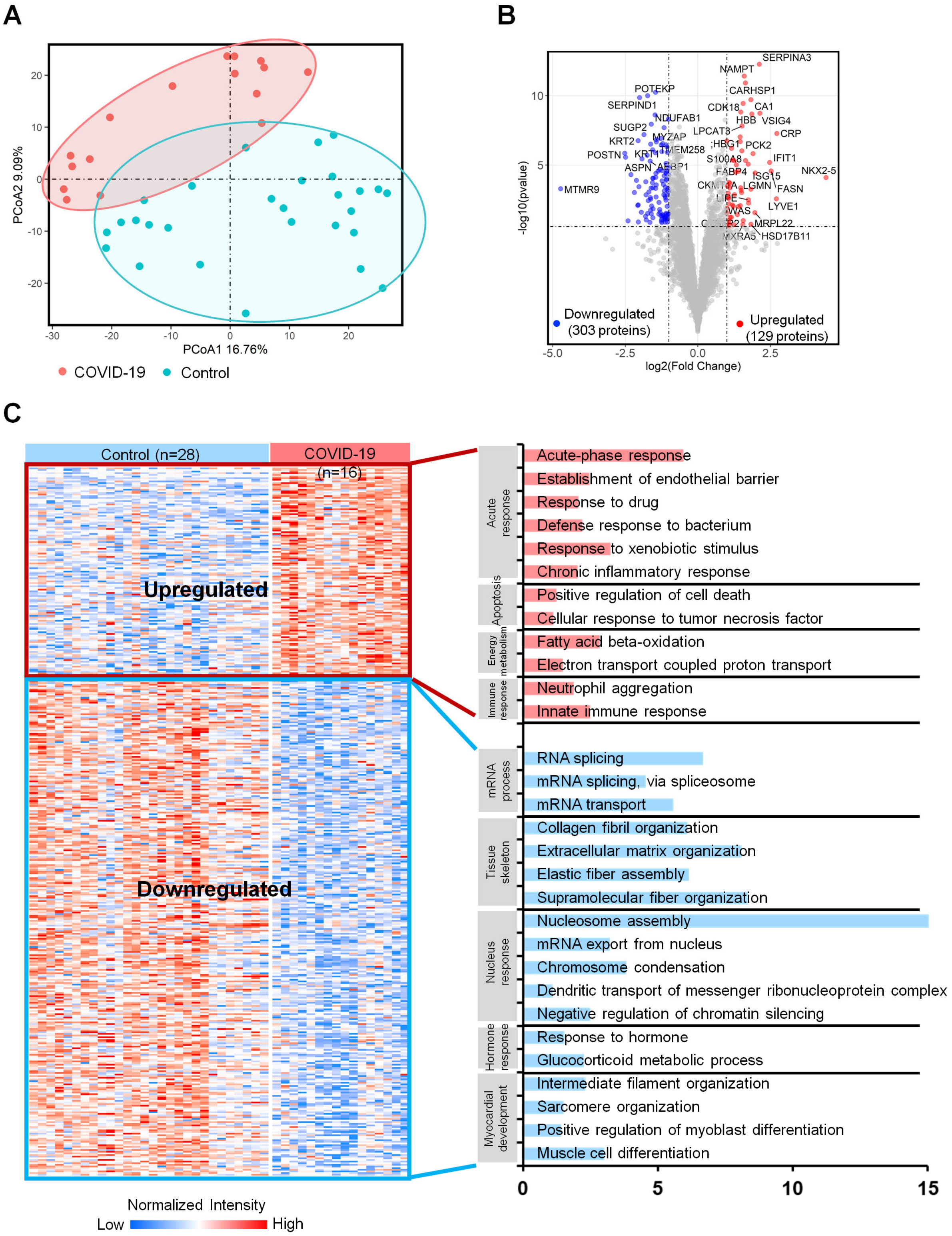

Figure S1. Quantitative proteome profiling of the SARS-CoV-2-infected heart tissues. (A) The PCoA of the COVID-19 patient and the control heart tissue samples revealed the existence of distinct groups. (B) Volcano plot of the $-\log_{10}$ of the *p*-value *vs*. the $\log_2$ of the protein abundance of DEPs after comparing COVID-19 patient heart tissue samples with control heart tissue samples; proteins outside the significance threshold lines are highlighted in red (upregulated DEPs) or blue (downregulated DEPs). COVID-19, coronavirus disease 2019; DEPs, differentially expressed proteins. (C) Heatmap showing the distinct patterns of DEPs in COVID-19 patient and control heart tissue samples; the functional annotation analysis of the DEPs of the COVID-19 patient and the control heart tissue groups was characterized based on the biological processes involved; the red and blue color bars represent the scaled expression levels of proteins in the two groups; the DEPs between the COVID-19 ($n = 16$) and the control ($n = 28$) samples were determined based on the Benjamini-Hochberg-adjusted *p*-value of a moderated *t*-test *p*-value that was <0.05, with a $\log_2$(COVID-19/control) > 0.585 (upregulated), and a $\log_2$(COVID-19/control) < -0.585 (downregulated).

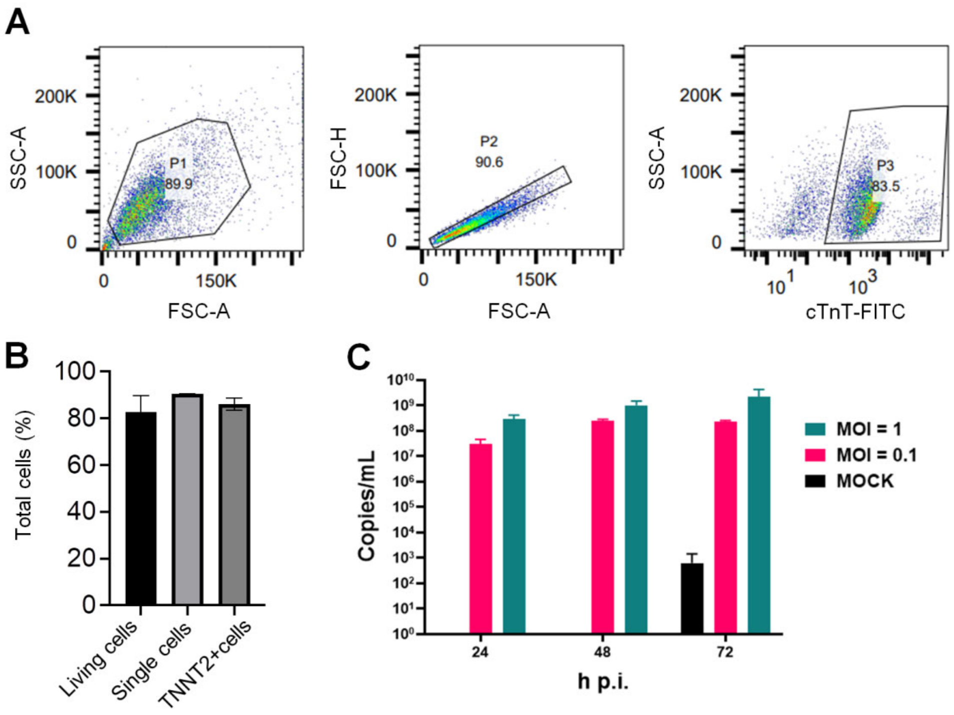

Figure S2. Evaluation of the purity of hiPSC-derived cardiomyocytes and SARS-CoV-2 MOIs. (A) Representative plots of flow cytometry showing cTNT expression in iPSC-derived cardiomyocytes. (B) Ratio of living cells, Single cells, and TNNT2+cells to the total cells with Flow cytometry gating analysis.(C) Evaluation of the SARS-CoV-2 MOI; given an MOI of 1 and of 0.1 as well as a control group consisting of mock (0 h), the copies of the virus at 24, 48 and 72 h post-infection (p.i.) were measured.

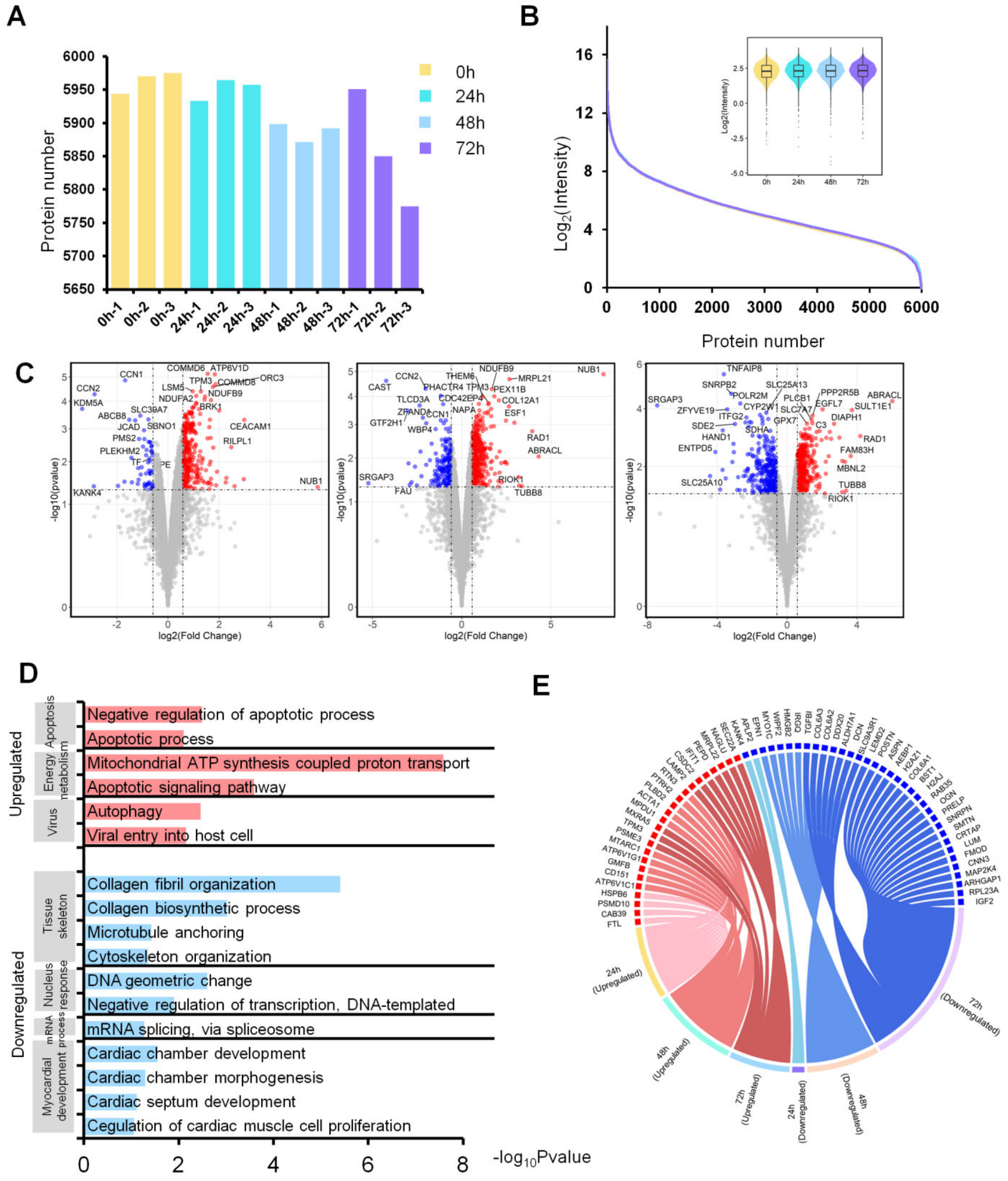

Figure S3. Proteins identified in SARS-CoV-2-infected hiPSC-derived cardiomyocytes at different timepoints post-infection. (A) Numbers of proteins identified in each sample at 0, 24, 48, and 72 h post-infection; a total of four timepoints was assessed, and each timepoint was

assessed through three biological repeats. (B) Intensity distribution of the identified proteins in each sample at all four assessed timepoints; the protein abundance of the samples was uniformly comparable across the different timepoints examined. (C) Volcano plot of the $-\log_{10}$ of the *p*-value *vs*. the $\log_2$ of the protein abundance of DEPs after comparing SARS-CoV-2-infected hiPSC-derived cardiomyocytes at 24, 48, and 72 h post-infection with the control samples; proteins outside the significance threshold lines are highlighted in red (upregulated DEPs) or blue (downregulated DEPs). (D) Functional annotation analysis of upregulated and downregulated-expressed proteins between SARS-CoV-2-infected hiPSC-derived cardiomyocytes and control samples. The DEPs between the infected (n = 3 for each timepoint post-infection) and the control (n = 3) samples were determined based on the Benjamini-Hochberg-adjusted *p*-value of a moderated *t*-test *p*-value that was <0.05, with a $\log_2$(Infected/Control) > 0.585 (upregulated), and a $\log_2$(Infected/Control) < −0.585 (downregulated). (E) Circos diagram shows the commonly DEPs in heart tissues and infected hiPSC- derived cardiomyocytes at different timepoints.
